# Tracking and Characterizing Spatiotemporal and Three-Dimensional Locomotive Behaviors of Individual Broilers in the Three-Point Gait-Scoring System

**DOI:** 10.3390/ani13040717

**Published:** 2023-02-17

**Authors:** Guoming Li, Richard S. Gates, Meaghan M. Meyer, Elizabeth A. Bobeck

**Affiliations:** 1 Department of Poultry Science, The University of Georgia, Athens, GA 30602, USA; 2 Institute for Artificial Intelligence, The University of Georgia, Athens, GA 30602, USA; 3 Department of Agricultural and Biosystems Engineering, Iowa State University, Ames, IA 50011, USA; 4 Department of Animal Science, Iowa State University, Ames, IA 50011, USA; 5 Egg Industry Center, Iowa State University, Ames, IA 50011, USA

**Keywords:** broiler, welfare assessment, behavior recognition, deep learning, tracking

## Abstract

**Simple Summary:**

Objective gait scoring can provide critical insight into broiler chicken welfare, including physiological traits related to leg health status. This research aimed to track and characterize spatiotemporal and three-dimensional locomotive behaviors of individual broilers using three gait scores typically used for evaluating bird walking ability in the U.S. broiler industry. Birds were placed on a custom-built platform and manually gait-scored while top-view videos and depth images were simultaneously recorded. A deep-learning-based interface was used to analyze videos and images, and then locomotive behaviors of individual broilers were analyzed from these extracted metrics. Results show that broilers with lower gait scores (less difficulty walking) presented more obvious lateral body oscillation patterns; further, forward (linear)-moving acceleration along the platform was significantly different for broilers across the three gait-score categories. Locomotive-behavior tracking and characterizing methods could be useful tools for automatically and objectively gait-scoring broilers, hence providing great support for precision broiler management.

**Abstract:**

Gait scoring is a useful measure for evaluating broiler production efficiency, welfare status, bone quality, and physiology. The research objective was to track and characterize spatiotemporal and three-dimensional locomotive behaviors of individual broilers with known gait scores by jointly using deep-learning algorithms, depth sensing, and image processing. Ross 708 broilers were placed on a platform specifically designed for gait scoring and manually categorized into one of three numerical scores. Normal and depth cameras were installed on the ceiling to capture top-view videos and images. Four birds from each of the three gait-score categories were randomly selected out of 70 total birds scored for video analysis. Bird moving trajectories and 16 locomotive-behavior metrics were extracted and analyzed via the developed deep-learning models. The trained model gained 100% accuracy and 3.62 ± 2.71 mm root-mean-square error for tracking and estimating a key point on the broiler back, indicating precise recognition performance. Broilers with lower gait scores (less difficulty walking) exhibited more obvious lateral body oscillation patterns, moved significantly or numerically faster, and covered more distance in each movement event than those with higher gait scores. In conclusion, the proposed method had acceptable performance for tracking broilers and was found to be a useful tool for characterizing individual broiler gait scores by differentiating between selected spatiotemporal and three-dimensional locomotive behaviors.

## 1. Introduction

Gait scoring is a critical technique for evaluating broiler leg health; it works by directly assessing a bird’s locomotive ability and assigning a numerical rating [1,2]. Due to a broiler’s gait being a direct reflection of its ability to access resources (e.g., feed and water) and socialize with its neighbor, gait scoring is associated with broiler production efficiency [3], welfare status [4], bone quality [5], physiology, and metabolism [6]. Trade associations and governmental agencies have realized the significance of gait scoring in the broiler industry and provided various recommendations. The National Chicken Council [7] stated that gait scoring must be performed on 100 birds, once per flock, no earlier than seven days prior to slaughter. The European Commission [8] listed gait score as an animal-based measure to assess a bird’s degree of musculoskeletal disorder (infectious, developmental, and degenerative).

The gait-score system developed by Kestin et al. [1] has been used to assess leg health in broilers; it is an empirical method consisting of visual observations of birds as they walk across a surface. The scoring system is divided into six categories of observed leg health status from completely normal to immobile. Despite being widely used on commercial farms, the six-point gait-scoring system has subtle differences between categories and requires well-trained assessors and substantial time for gait assessment. Garner et al. [9] simplified gait scoring into four categories by removing the intermediate scores that are perhaps more challenging to estimate; however, this system also faces the same issue of subjectivity on intermediate scores as the six-point system. Accordingly, Webster et al. [10] developed and validated a three-point gait-scoring system for assessing the walking ability of commercial broilers in the United States [7]. This three-point system distinguishes among broilers with no impairment of walking ability (score 0), obvious impairment but still ambulatory (score 1), and severe impairment and being unable to walk without great difficulty (score 2). Although the three-point system improved assessment agreement and correlation between observers in identifying lame birds, it still involves a degree of subjectivity from the manual gait scoring by observers.

Appropriate assessment protocols are critical for accurately and efficiently quantifying broiler gait scores. The most commonly used protocol is to manually observe individual birds walking and then score them [1,9,10], a time-consuming process prone to human errors. In principle, an automated computer-vision-based scoring system could provide an objective, efficient, and accurate assessment. Aydin et al. [11] compared pixel changes between frames to calculate a bird activity index and reported a significant relation between gait scores assessed by experts and activities monitored by image analysis. Dawkins et al. [12] provided descriptive statistics (mean, variance, skewness, and kurtosis) of the optical flow of ten intensively housed commercial broiler flocks between the ages of 32 and 35 days. The results showed that all four measures were significantly correlated with the percentage of birds with poor locomotive behavior and, therefore, a high gait score. Silvera et al. [13] derived features from an activity index before and after a human assessor walked through the flock and used the features to predict gait scores of broilers. These automated systems recognized group-level gait scores but could not assess an individual bird’s gait score.

Individual broiler monitoring may be expensive and technically difficult for commercial production barns with tens of thousands of broilers of similar appearance in a group setting, but it can provide detailed and valuable information for laboratory-scale broiler experiments [14]. van der Sluis et al. [15] used an ultrawideband tracking system to characterize the activity of individual broilers and found significant differences in average moving distances and other derived metrics between broilers with good and suboptimal gait scores. The system required having battery-powered tags attached to each broiler, which could be expensive for large-scale experiments. Aydin developed RGB- and depth-based computer vision systems for capturing locomotive behaviors of individual broilers placed in a corridor [16,17]. The step frequency, step length, step speed, lateral body oscillation, and latency to lie were significantly different among broilers with various gait scores [16]. However, the three-point gait-scoring system currently used in U.S. broiler industry [7] has not been used in this context. Introducing three-dimensional and spatiotemporal measures to automatically implement a three-point scoring system may allow for more straightforward adoption. Meanwhile, the abovementioned studies mainly applied conventional image processing algorithms for behavior recognition, whereas recent state-of-the-art deep-learning techniques generally outperformed conventional image processing [18]. The accuracy and reliability of these ideas need to be strictly validated in the context of broiler gait scoring.

The study objective was to track and characterize spatiotemporal and three-dimensional locomotive behaviors of individual broilers with different gait scores, as measured by the three-point gait-scoring method. Deep-learning algorithms, depth sensing, and image processing were jointly deployed, developed, and validated for behavior recognition.

## 2. Materials and Methods

### 2.1. Housing, Animals, and Management

The experiment was conducted in the Robert T. Hamilton Poultry Teaching and Research Facility at Iowa State University (Ames, IA, USA) for a 7-week grow-out period. A total of 1360 straight-run Ross 708 broiler chicks were obtained from a commercial hatchery (International Poultry Breeders Hatchery, Bancroft, IA, USA) and randomly distributed to identical floor pens. Five birds per pen from 14 pens were selected as focal birds for gait scoring. Each pen contained 34 birds and was 2.44 m long and 1.22 m wide. The experiment was conducted from January to March 2022. Approximately 10 cm deep fresh wood shavings provided bedding over the solid concrete floor, and PVC pipe dividers with mesh walls (1.22 m high) separated pens. Ambient temperature (°C, mean ± standard deviation) from the starter, grower, finisher 1, and finisher 2 periods was 30.64 ± 2.94, 25.23 ± 1.67, 24.22 ± 1.33, and 22.22 ± 3.11, respectively. Indoor relative humidity (%, mean ± standard deviation) from starter, grower, finisher 1, and finisher 2 periods was 21 ± 7.5, 25.8 ± 10.2, 27.4 ± 10.6, and 30.8 ± 5.6, respectively. Lighting was gradually adjusted from 24 L:0 D on day 0 to 20 L:4 D on days 8–42, and light intensities at bird level were reduced from 30–40 lux on day 0 to 20–30 lux for days 8–42. Chicks were brooded with two heat lamps per pen (22.9 cm reflectors with porcelain socket) using 125 W incandescent heat bulbs (Sylvania, Wilmington, MA, USA) for the first 9 days. Birds were fed ad libitum with diets formulated for Ross 708 commercial recommendations, with two hanging feeders (BRHF151, Brower Equipment, Houghton, IA, USA) per pen, gradually raised to accommodate bird height. Diets were fed for two weeks each, in 3 phases as a starter, grower, and finisher 1 and for one week for finisher 2, for the 7-week study. Full diet specifications are found in [19]. Water was provided ad libitum from a hanging nipple water line (4 nipples per pen). All live-bird procedures were approved by the Iowa State University Institutional Animal Care and Use Committee (IACUC #19-322, approval November 2021).

### 2.2. Gait Scoring and Data Acquisition

A subset of 70 broilers (5 birds per pen from 14 pens) was randomly assigned on day 0 as focal birds and manually gait-scored on a 3-point scale on days 38 and 45. These focal birds were marked with livestock spray paint (red, blue, green, pink, and orange). For manual gait scoring, birds were placed on a custom-designed plywood platform (1.8 m long, 0.46 m wide, and 0.30 m high). The platform has 0.15 m start and finish sections on each end of a 1.5 m long walking space, with delineations marked every 0.30 m. Focal birds were individually placed on the platform start section and either walked 1.5 m independently or were encouraged to walk by a researcher waving behind and gently tapping the bird with a ping-pong paddle. A bird was encouraged to move when seeing another bird placed on the other side of the platform. There were no time-out criteria for the gait scoring, and the time needed for gait-scoring birds in each category was uncertain. Birds were considered to have completed the gait-scoring session when both feet had crossed into the finish section. Following the guidelines of the three-point gait-scoring system from the creator [10] and the National Chicken Council [7], each bird was assigned with a score from 0–2: score 0 indicating the ability to walk 1.5 m with no signs of lameness, score 1 indicating the ability to walk 1.5 m with signs of lameness (obvious unevenness in steps or sitting down at least once), or score 2 indicating inability to walk 1.5 m. All birds were scored by the same individual.

A webcam (V-U0040 Webcam, Logitech International S.A., Lausanne, Switzerland) and RGBD camera having four channels of red, green, blue, and depth (Intel^®^ RealSense^TM^ Depth Camera D435, Intel Corporation, Santa Clara, CA, USA) were installed ~2.39 m above the platform to capture top-view videos and images, respectively. Videos were simultaneously recorded, with files stored in AVI format every 20 min, at a resolution of 1920 × 1280 pixels, and a sampling rate of 30 frames per second (fps). RGB and depth images were separately recorded with a resolution of 640 × 480 pixels, at a sampling rate of 10 fps. Birds required anywhere from a few seconds to several minutes to walk through the platform, and all videos/images were analyzed. The RGB and depth images at each timestamp were self-calibrated to ensure that spatial information of the two types of images was the same. All recordings were programmed using the open-source Python-based library, OpenCV. Two PCs were used, one (Intel^®^ Core^TM^ i7-4770 CPU @ 3.4 GHz processor, 8.0 GB installed RAM, and 64-bit Windows 10 operating system) for recording videos and the other (Intel^®^ Core^TM^ i5-6500 CPU @ 3.2 GHz processor, 8.0 GB installed RAM, and 64-bit Windows 10 operating system) for recording RGB and depth images. 

### 2.3. Overall Process

**Figure 1:**
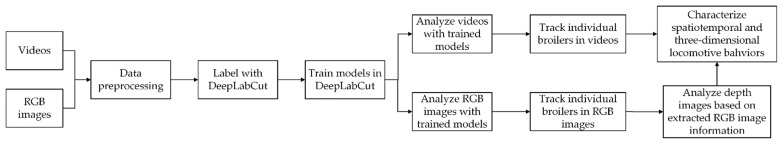
Overall process for tracking and characterizing spatiotemporal and three-dimensional locomotive behaviors of broilers.

The overall process of tracking and characterizing behaviors of interest was shown in Figure 1. Collected videos and RGB images were first preprocessed to remove unnecessary regions, then labeled using a deep-learning-based graphical user interface (GUI), DeepLabCut [20,21], with an assigned key point on each bird’s back. A deep-learning model within DeepLabCut was trained, and then videos and RGB images were analyzed by this model to extract the key point from image sequences of each broiler’s back. Next, an embedded tracking algorithm of the DeepLabcut package tracked individual broilers via their associated detected key points between adjacent frames of videos or RGB images. The depth information of the key points was obtained by linking the depth images with correlated spatial information in RGB images. Extracted video and depth information were used to characterize spatiotemporal and three-dimensional broiler locomotive behaviors. The image processing package in Python and DeepLabCut are compatible with RGB-type files, but depth information was required to obtain the three-dimensional locomotive behaviors; therefore, depth images were processed based on the extracted RGB image information. The lower-resolution RGB images captured by the RGBD camera may complicate the analysis procedures and not be further used to analyze the spatiotemporal behavior. Detailed explanations of each processing step are presented in the following sections. 

### 2.4. Data Preprocessing

The video and image files with bird locomotive behaviors and gait scores were manually selected. A total of four video episodes were randomly selected for each gait score, and each episode lasted from 0.5 to 3.5 min. Only four birds with a score of 2 were detected, and thus, an equal number of broilers with scores of 0 and 1 was used to match for statistical analysis. 

**Figure 2:**
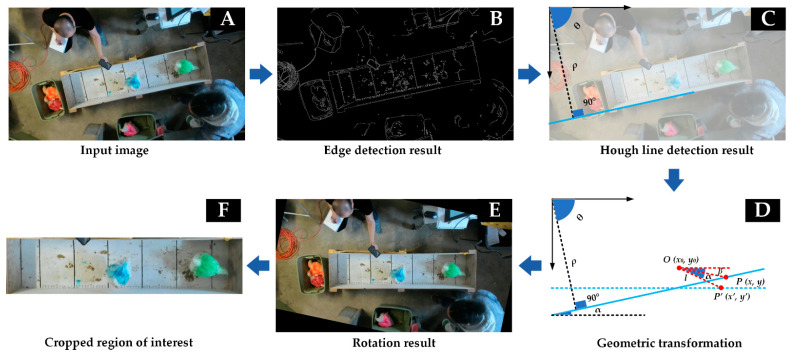
Workflow of the image preprocessing of RGB and video-frame images. (**A**) is the original image. (**B**) is the platform edge detection. (**C**) is the Hough line detection result. The solid blue line is the line detected by the Hough transform approach, from which the distance and angle needed for transformation are obtained. (**D**) is the geometric transformation. The dotted blue line is the rotated edge line, and the dotted black and red lines are auxiliary lines for calculation. ρ and θ are the radius and angle in the polar coordinate system and detected by the Hough transform approach, α is the angle for rotation, and β is the auxiliary angle for calculation. $\left(x_{0},y_{0}\right),\;\left(x,y\right),\;and\;\left(x^{\prime },y^{\prime }\right)$ are the coordinates of the center point *O* of an image, example point on the detected straight line *P*, and example point on the rotated straight line *P’*, respectively. (**E**) is the resultant rotational transform of the image, and (**F**) is the rotated and cropped region of interest for analysis.

Each frame in a video and RGB image was first preprocessed. The images of the walking platform included nonessential information and were rotated. Accordingly, the image was rotated to align the bottom edge of the platform with that of the image. All other unnecessary regions were removed so that bird movement appeared as mostly horizontal. This process is illustrated in the series of images in Figure 2, which depicts the steps taken from an original overhead image (Figure 2A) to a final image for analysis (Figure 2F). The rotated platform’s edges in the input image (Figure 2A) were detected using a common edge-detection algorithm, Canny Algorithm [22] (Figure 2B). The algorithm found points with maximal gradient magnitude in an image and connected the detected points to form an edge using step edge detectors and elongated operators. Then, straight lines (assuming the sides of the platform to be straight lines in an image) were detected using Hough Transform [23] (Figure 2C). The Hough algorithm transformed all coordinates $\left(x_{i},y_{i}\right)$ in the image into a polar coordinate system, with the top-left corner serving as the origin of an image. All straight lines fitted into Equation (1) were extracted along with a specific set of the radius ρ and angles θ. The line (solid blue line, Figure 2D) with the highest ranking in a set of detected straight lines corresponded to the long side of the platform and was used as a reference to correct the rotated platform image.

(1) $$\rho =x_{i}\cos\theta +y_{i}\sin\theta$$

where ρ and θ are the radius and angle in a polar transform system, respectively. θ is the detected degree by an algorithm and converted to the degree of rotation needed between original and rotated images.

Additional geometric transformation was needed for image rotation. Given the radius ρ and angle θ (Figure 2D), the rotation angle α was calculated as follows:

(2) α=90°−θ

(3) $$\left[\begin{array}{c} x^{\prime }\\ y^{\prime } \end{array}\right]=\left[\begin{array}{ccc} cos\alpha & -sin\alpha & x_{0}\left(1-cos\alpha \right)+y_{0}sin\alpha \\ sin\alpha & cos\alpha & -x_{0}sin\alpha +y_{0}\left(1-cos\alpha \right) \end{array}\right]\left[\begin{array}{c} x\\ y\\ 1 \end{array}\right]\;$$

where $\left(x^{\prime },y^{\prime }\right)$ and (x,y) are the coordinates before and after the rotation. $\left(x_{0},y_{0}\right)$ is the centroid coordinate of the original image. 

The entire image was rotated based on Equations (1)–(3), and the blank regions after rotation were filled with black pixels to maintain the same dimensional scale as the original image. In the rotated image, the top-left coordinates $\left(x_{min},y_{min}\right)$ and bottom-right coordinates $\left(x_{max},y_{max}\right)$ of the platform were used to crop all but the region of interest (platform). The data preprocessing steps were executed for all videos and RGB images. 

### 2.5. Deep-Learning Modeling and Development

The cropped videos and RGB images were input into a deep-learning-based graphical user interface (GUI), DeepLabCut [20,21], for further processing. DeepLabCut is a multifunctional interface that can label key points on a frame, train deep-learning models to detect key points, and track detected key points throughout a video. Videos were processed to extract the center key point of each broiler’s back; this key point was used to project broiler locomotive behavior across time. Key points in the depth images were found based on the detected coordinates from the RGB images/videos. The pixel-to-distance conversion factor for calculating the RMSEs and movement trajectories (Section 2.6) was estimated as 0.62 pixels∙mm^−1^. 

**Figure 3:**
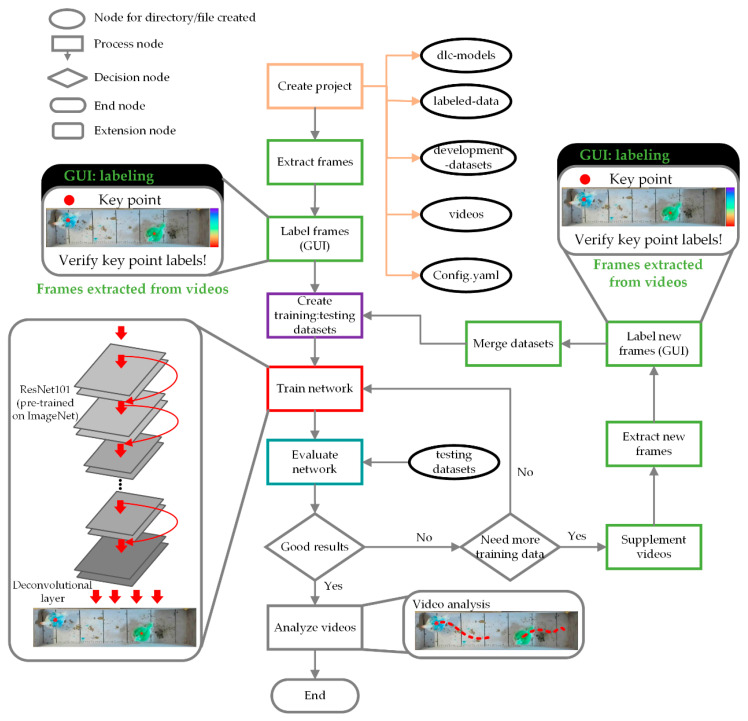
Flowchart for video processing using the deep-learning framework, DeepLabCut. Dlc is the abbreviation of DeepLabCut, and GUI is graphical user interface. Red arrows indicate the directions of processing.

An overview flowchart of the DeepLabCut modeling and development is indicated in Figure 3. A total of 60 frames were extracted from each video using the K-nearest-neighbor algorithm, and then the key point on a broiler’s back from the extracted frames was labeled with DeepLabCut. A deep-learning model, ResNet101 [24], was trained to recognize the key point on the back. Training loss was continuously reported and visualized. The ratio of training to testing images was set to 80:20. A training sequence involved starting with a select dataset until training loss performance stalled, and then more image samples were supplemented to the dataset and the process repeated until optimal loss performance was achieved. Accuracy and root-mean-square error (RMSE) were used to assess the final performance of the trained algorithm. Videos were analyzed with broilers abstracted as key points using the well-trained model. 

### 2.6. Locomotive-Behavior Metric Definitions and Calculation

**Figure 4:**
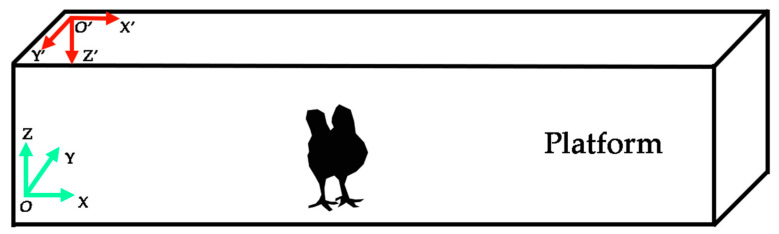
Coordinate systems for broiler locomotive-behavior analysis. O’-X’-Y’-Z’ is the image coordinate system, and O-X-Y-Z is the Cartesian coordinate system used for bird motion.

The locomotive-behavior metrics were analyzed based on the extracted coordinates of the key point for each bird, as mentioned in Section 2.5. All extracted coordinates were converted from the image coordinate system to a Cartesian coordinate system for behavior analysis (Figure 4). Bird trajectories were depicted with extracted coordinates along the XY plane of the Cartesian system for describing horizontal movement situations and along the YZ plane for displaying vertical movement scenarios.

Moving distances between adjacent frames were multiplied by the frame rate of video acquisition (30 fps) to obtain linear moving speed (mm∙s^−1^) in the X and Y directions and multiplied by the frame rate of depth image acquisition (10 fps) to obtain the speed in the vertical (Z) direction. Similarly, the linear moving acceleration (mm∙s^2^) along the three axes was acquired by dividing the difference in linear moving speed between adjacent frames with respective time intervals. The linear moving speed and acceleration were calculated for each bird, and they were transformed to positives via the absolute operator for statistical analysis, for which only the absolute movement degree was compared. 

Forward-moving locomotive behavior was defined as a bird moving along the *X*-axis and can directly reflect a bird’s walking ability. Only the forward-moving duration (s∙bout^−1^) during each moving event was calculated, because birds could be encouraged to move via human manipulation, and the results of other forward-moving locomotive behaviors (e.g., forward-moving bouts and forward-moving duration along the platform) could be interfered with. The calculation procedure of forward-moving duration per bout is presented in pseudocode form as Algorithm 1. The program compared the moving distance between adjacent frames (with a time interval of 1/30 s), and birds were defined as being forward-moving when the distance was more than 5 mm, which was the minimum threshold based on the validation of a moving bird from the top-view videos. Some electronic noise in the images could also result in an instantaneous distance change between adjacent frames, and this noise could be filtered if a duration for the change was set. Therefore, if birds continued to move over 3 frames (~0.1 s), the forward-moving duration was stored for further processing.**Algorithm 1: Calculating forward-moving duration per bout**
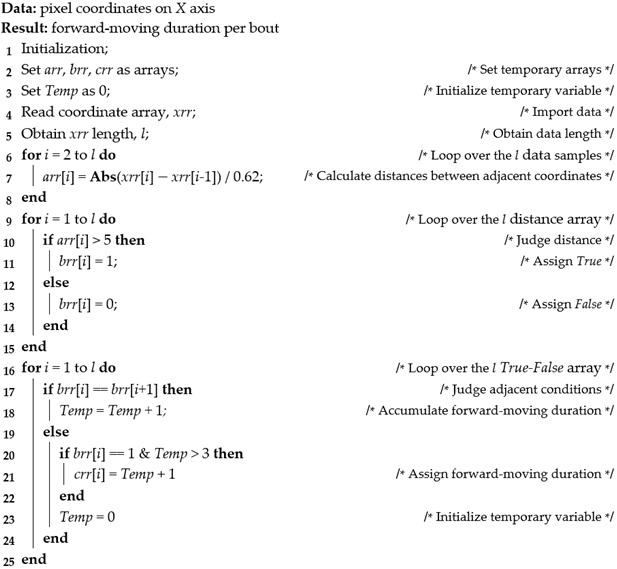


Rapid-growing broilers have been observed to have a tendency to waddle, that is, to walk with a degree of lateral body oscillation, presumably due to underdeveloped skeletal structure (especially within the legs) and an uneven body center of gravity [25]. The centroid on these broilers’ backs could be seen to oscillate periodically along the *Y*-axis, and some birds would rest intermittently and did not always continuously complete a whole cycle of lateral body oscillation. Therefore, half-cycle lateral body oscillation durations were calculated (Algorithm 2). Similar to the calculation of forward movement along the *X*-axis, the lateral body movement in the Y direction was counted if the moving distance exceeded 5 mm between adjacent frames and the movement lasted over 3 frames (0.1 s). The half-cycle of lateral body oscillation should contain a set of positive moving distances and another set of negatives in the Y direction.**Algorithm 2: Calculating half-cycle duration of lateral body oscillation**
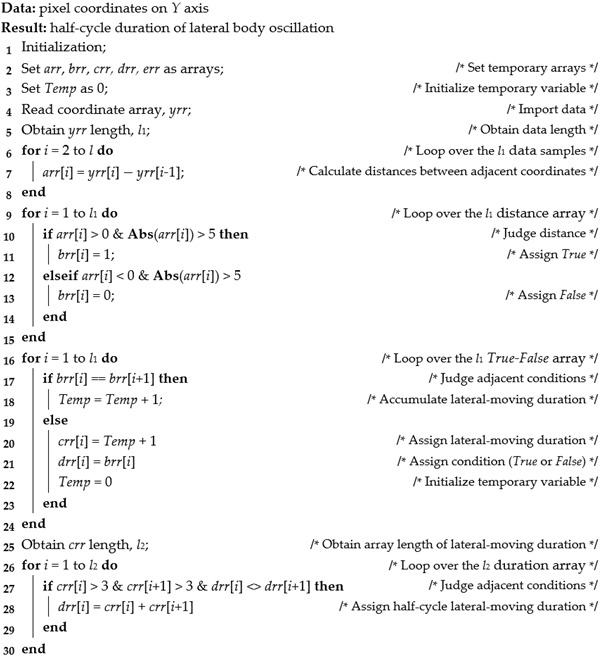


These locomotive behaviors were continuously analyzed for each bird in each detected video episode via DeepLabCut, and the average and maximum of these behaviors were extracted for further analysis. 

### 2.7. Statistics 

The relationship between lameness as reflected by gait scores and the average and maximum of linear moving speed, linear moving acceleration, forward-moving duration per bout, and half-cycle duration of lateral body oscillation was examined by employing a one-way ANOVA using the PROC MIXED statement in the Statistical Analysis Software (SAS 9.3; SAS Institute Inc., Tokyo, Japan). The fixed effect in the statistical model was lameness, reflected by gait scores. The experimental unit was a broiler. Least-square mean comparisons of the behaviors were conducted using Fisher’s least-significant difference with PDMIX800 [26], with significance considered at *p* ≤ 0.05. The results were presented as mean ± pooled standard error. 

## 3. Results

### 3.1. Deep-Learning Model Performance

**Figure 5:**
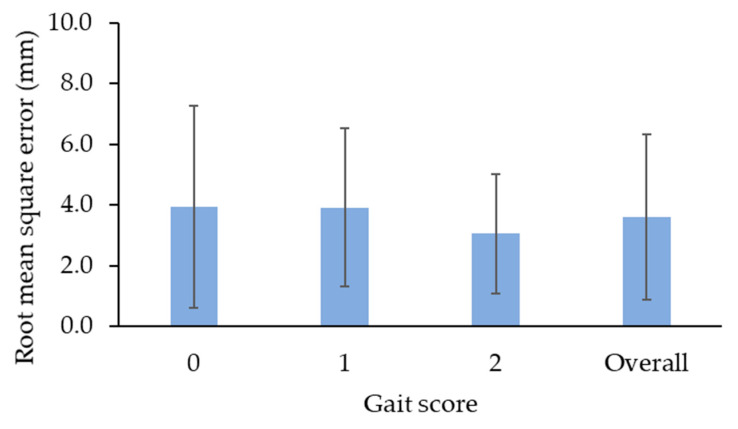
Root-mean-square error of key points on broilers’ backs between ground truth and prediction.

The ResNet101 achieved the best training performance at the 200,000th iteration, with a training loss of 0.0002, and thus, the model at that iteration was saved for testing and video analysis. The DeepLabCut embedded with the trained ResNet101 model achieved 100% accuracy in recognizing key points on the back of a broiler on the platform. The average RMSE was 4.0 ± 3.3 mm for score 0, 3.9 ± 2.6 mm for score 1, and 3.1 ± 2.0 mm for score 2 (Figure 5), with an overall RMSE of 3.6 ± 2.7 mm. These all indicate that the trained deep-learning-based platform could accurately identify the key points and was appropriate for video analysis in this case.

### 3.2. Three-Dimensional Trajectories of Broilers in the Three-Point Gait-Scoring System

**Figure 6:**
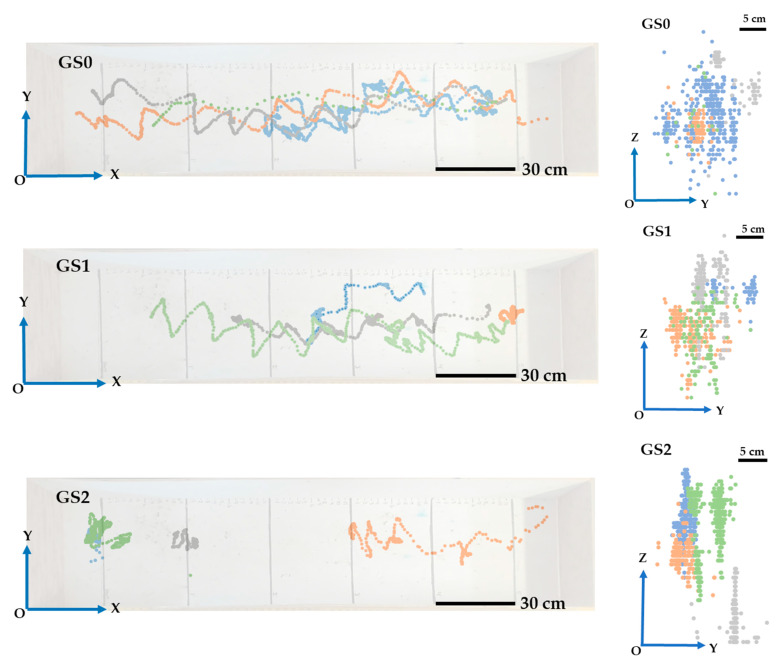
Movement trajectory of broilers with different gait scores. (**Left**) on XY plane; (**Right**) on YZ plane.

The three-dimensional movement trajectories of the 12 birds with the three levels of gait scores are presented in Figure 6. On the XY plane, the zigzag walking patterns (transverse oscillation along the forward axis) were observed for broilers with gait scores of 0 and 1 more often than for broilers with a score of 2, and the zigzag trajectory length of broilers with a gait score of 0 was longer than those with scores of 1 and 2. The zigzag walking pattern is associated with lateral body oscillation. Despite having no severe leg problems, some birds, even with human encouragement, were reluctant to move, resulting in broilers with scores of 0 and 1 being unable to finish the 1.50 m long walking assignment. Birds with severe leg problems (score of 2) were most likely to stay at the initial location where they were placed or waddle without standing up on the platform. On the YZ plane, the narrow movement trajectories were observed for broilers with a score of 2 but were not obvious for scores of 0 and 1. Lame birds (score of 2) attempted to stand up to walk with human encouragement but mostly failed and sat down, resulting in the clear up-and-down movement pattern along the *Z*-axis. 

### 3.3. Moving Speed of Broilers in the Three-Point Gait-Scoring System

Table 1 compares three-dimensional linear moving speed and acceleration for broilers with three gait scores. The moving speed ranged from 8.3 to 368.3 mm∙s^−1^, 7.3 to 71.5 mm∙s^−1^, and 26.5 to 150.5 mm∙s^−1^ in the directions of X, Y, and Z, respectively. The linear moving acceleration was 5.6 to 154.2 mm∙s^−2^, 6.6 to 67.2 mm∙s^−2^, and 26.0 to 140.0 mm∙s^−2^ in the X, Y, and Z directions, respectively. The average and maximum of the linear moving speed and the average of the linear moving acceleration in the X and Y directions for a score of 0 were significantly or numerically higher than those for a score of 1, and except for the average of the linear moving acceleration, those parameters for a score of 1 were just numerically higher than those for a score of 2. There was no significant difference for the maximum of the linear moving acceleration in the directions of the X and Y axes (*p* ≥ 0.17), while the linear moving speed and acceleration were not significantly different along the *Z*-axis (*p* ≥ 0.32). 

### 3.4. Moving Duration of Broilers in the Three-Point Gait-Scoring System

Table 2 presents the forward-moving duration, i.e., the duration of motion increments in the X direction and half-cycle lateral body oscillation along the *Y*-axis. While the irregular vertical movement was observed, its movement duration was not analyzed. The forward-moving duration was 0.15 to 1.27 s∙bout^−1^, and half-cycle duration of lateral body oscillation was 0.37 to 1.48 s. The average and maximum of the forward-moving duration for a gait score of 0 were significantly higher than that of a score of 1, while the two metrics of half-cycle duration of lateral body oscillation were similar between the two scores. Except for the maximum of half-cycle duration of lateral body oscillation, the other metrics of the moving duration for a score of 1 were similar to that for a score of 2.

## 4. Discussion

This project aimed to track and characterize three-dimensional and spatiotemporal locomotive behaviors of broilers gait-scored with the three-point gait-scoring system. The trained deep-learning-based platform, DeepLabCut, achieved extremely good key point identification and tracking performance with 100% recognition accuracy and 3.62 ± 2.71 mm estimation deviation between ground truth and prediction. This performance relied on multiple factors. Birds were marked with five colors (red, blue, green, pink, and orange) so that they could be easily identified within pen flocks by caretakers. The distinct color features on the back of the broilers may assist in the recognition performance. Similarly, Toshiyuki et al. [27] also sprayed various colors on the backs of broilers to promote the bird-tracking performance via image processing. It should be noted that the coloring method may be favorable for laboratory broiler experiments but is not suitable for commercial application because of the sheer number of birds in a house. The careful design of the image preprocessing steps removed unnecessary background information and cropped the region of interest, facilitating the key-feature (back of a bird) recognition. The unsupervised machine-learning algorithm, KNN, selected the most challenging frames in video episodes. The selected frames contained diverse and sufficient features and variations for a model to learn, thus increasing the inference performance [21]. 

Bird shape can be deformed while birds are walking. However, the cameras were installed in close proximity to the platform and birds (~2.39 m above the platform), and the high resolution of the images and detailed features of the birds can be captured, resulting in the small root-mean-square error of 3.6 ± 2.7 mm for localizing individual birds. Therefore, there were no significantly inaccurate results for depicting the overall trajectories of individual birds as affected by bird shape deformation during walking. Although only one key point was picked, it is representative. The selected key point was on the back of each bird, which is nearly the center of a bird, providing a relatively stable metric for overall trajectory description. The points on the head, wing, and tail of a bird can be changed and deformed more frequently as the bird performs various active behaviors (e.g., head/tail shaking and wing flapping); therefore, they were not optimal for the key-point detection. Including multiple key points into animal trajectory remains an investigation and exploration for future research.

Modern broiler chickens possess an unbalanced body conformation due to genetic selection for fast-growing breast muscle and body mass, which promotes compensatory gait and behavior adaptations (e.g., lateral body oscillation) to minimize energy expenditure [28]. Caplen et al. [28,29] reported that lame broilers often walk with greater lateral body oscillations than non-lame birds. In contrast, our study found lame broilers (score 2) to have barely any lateral body oscillation, mostly because they instead sat down or waddled on the platform rather than attempting to walk upright. A compromised leg structure cannot fully support the disproportionately large upper body of a lame bird. Perhaps the more obvious lateral body oscillation patterns may indicate better walking ability of broilers at the end of a production cycle. 

The overall behavior metrics were reasonably comparable to those reported in previous studies. The linear moving speed of broilers at 38–45 days of age in this study was comparable to 74–166 mm∙s^−1^ for 39-day-old broilers [17] and slower than 710.0–1006.5 mm∙s^−1^ for 32-day-old broilers [28]. Older broilers manifested poorer locomotive ability [30]. The linear moving acceleration in this study was much higher than the 0–30 mm∙s^−2^ reported by de Alencar Nääs et al. [31]; however, the floor surfaces were very different, with an 8 cm deep rice hull litter used by de Alencar Nääs et al. [31] compared to the litter-free flat lumber used in the present study. Although the former material was favorable to simulating bird-rearing situations, it could also prevent birds from accelerating quickly. Li et al. [14] reported that 87.4–92.0% of 28-day-old broilers continuously walked around a feeder for less than 2 s during each walking event, which is higher than the forward-moving duration per bout in this study. Younger birds could move longer per bout with a lighter body-mass burden, and feed attraction may also motivate broilers to walk longer around the feeder. The half-cycle duration of lateral body oscillation was comparable to the 0.6–1.4 s reported by Reiter and Bessei [25]. 

The moving-speed trends among the gait scores were in line with previous studies, which found that broilers with lower gait scores moved and accelerated faster than those with higher gait scores. Despite using various gait-scoring systems, de Alencar Nääs et al. [31] also elaborated that Ross 308 female broilers decreased in velocity from 57.2 mm∙s^−1^ for a score of 0 to 2.1 mm∙s^−1^ for a score of 4, and in acceleration from 1.9 mm∙s^−2^ for a score of 0 to 0.0 mm∙s^−2^ for score of 4. There is no research studying the forward-moving or walking duration per bout for broilers with different gait scores, but Reiter and Bessei [25] reported that the whole-cycle duration of lateral body oscillation was 1.2 ± 0.2 s for broilers without leg disorders and 4.0 ± 1.4 s for broilers with leg disorders, which is an opposing outcome to this study. The lateral body oscillation in this study was only accounted for when the program detected that the lateral moving distance changed over 5 mm between adjacent frames, and the changes lasted for over three frames (0.1 s). Although the program provided an objective measure of the behavior, it did exclude intermittent resting time, which typically appeared when lame birds walked along the platform, resulting in a lower half-cycle duration of lateral body oscillation for broilers with higher gait scores. 

Of the sixteen different locomotive-behavior metrics, only the linear moving acceleration in the X direction was significantly different among gait scores, suggesting its utility as a metric to differentiate broilers with three levels of gait scores. Similarly, Aydin [16,17] measured the number of lying events, latency to lie down, step frequency, step length, speed, and lateral body oscillation, and only the latency to lie down was significantly different among gait scores of 0 to 4. The behaviors and feature variables were quite similar between adjacent gait scores. New feature variables should be inserted into the proposed system for more accurate identification of the effects of lameness reflected by gait scores on the locomotive behaviors of broilers [17]. Alternatively, the proposed system could be combined with other automatic behavior-analysis systems, such as accelerometers and sound-recognition systems, to boost the performance of recognizing broilers with different gait scores [32]. It is worth noting that because there were very few lame birds in this experiment, the resultant sample size (*n* = 4 per gait score) was quite small. Curiously, some birds without leg disorders (score of 0) were reluctant to move, even though the assessor encouraged them; this may have led to increased similarities in the locomotive behaviors between the birds with and without leg disorders. Splitting the dataset only into training and testing sets without a validation set may result in crossover between data sources and the inability to tune hyperparameters due to small amounts of images and videos from small sample sizes, possibly leading to model overfitting (indicated by 100% accuracy in the study). The overfitting might be favorable for precisely analyzing current small sets of videos and images, but the developed algorithm was not suitable for being extended to other projects with more samples and variations [17]. More samples should be examined in the future. 

## 5. Conclusions

Spatiotemporal and three-dimensional locomotive behaviors of broilers with three levels of gait scores were comprehensively tracked and characterized. Deep-learning algorithms, depth sensing, and image processing were jointly developed and validated for the behavior recognition. The results show that the trained deep-learning-based platform, DeepLabCut, achieved 100% accuracy and 3.62 ± 2.71 mm RMSEs on estimating a key point on the back of broilers. Broilers with lower gait scores showed more obvious lateral body oscillation patterns and moved faster and longer in each movement event than those with higher gait scores.

## Figures and Tables

**Table 1:** Linear moving speed and acceleration for broilers with three gait scores.

Axis	Gait Score	Linear Moving Speed (mm∙s^−1^)		Linear Moving Acceleration (mm∙s^−2^)	
		Average	Maximum	Average	Maximum
X	0	233.6 a	368.3 a	84.5 a	154.2
	1	23.1 b	118.5 b	22.3 b	104.9
	2	8.3 b	72.9 b	5.6 c	67.2
	s.e.	64.4	56.8	6.9	29.5
	*p*-value	0.04	0.01	<0.01	0.17
Y	0	40.0 a	82.1 a	38.5 a	67.2
	1	17.2 b	71.5 ab	17.3 ab	60.6
	2	7.3 b	44.7 b	6.6 b	39.7
	s.e.	5.0	10.8	8.1	10.3
	*p*-value	<0.01	0.04	0.05	0.20
Z	0	26.5	70.0	32.7	62.3
	1	26.6	150.5	27.6	140.0
	2	30.5	88.0	26.0	66.5
	s.e.	4.5	42.2	8.0	38.3
	*p*-value	0.78	0.40	0.83	0.32

^abc^ Values within the same column that lack a common letter differ significantly (*p* ≤ 0.05). s.e. is pooled standard error.

**Table 2:** Forward-moving duration and half-cycle duration for lateral body oscillation.

Gait Score	Forward-Moving Duration (s∙bout^−1^)		Half-Cycle Duration of Lateral Body Oscillation (s)	
	Average	Maximum	Average	Maximum
0	0.73 a	1.27 a	0.86 a	1.48 a
1	0.23 b	0.33 b	0.66 ab	1.15 a
2	0.15 b	0.18 b	0.37 b	0.68 b
s.e.	0.21	56.8	0.11	0.21
*p*-value	0.01	<0.01	0.03	0.02

^abc^ Values within the same column that lack a common letter differ significantly (*p* ≤ 0.05). s.e. is pooled standard error.

## Data Availability

Not applicable.
